# Understanding Right Heart Flow: Implications for Interatrial Shunt Device Therapy in Heart Failure

**DOI:** 10.1016/j.jscai.2024.102439

**Published:** 2025-01-21

**Authors:** Raviteja Guddeti, Pankaj Garg, Dean J. Kereiakes, João L. Cavalcante, Marcus Carlsson, Santiago Garcia

**Affiliations:** a The Carl and Edyth Lindner Center for Research and Education at The Christ Hospital Network, Cincinnati, Ohio; b Norfolk and Norwich University Hospital, Norwich, United Kingdom; c Allina Health Minneapolis Heart Institute, Minneapolis, Minnesota; d Karolinska Institute, Solna, Sweden

**Keywords:** heart failure, interatrial shunts, magnetic resonance imaging, right heart flow

## Abstract

Elevation in left atrial pressure with subsequent pulmonary congestion is central to the pathology of heart failure. Interatrial shunts have emerged as a potential therapeutic strategy in patients with heart failure, especially those with diastolic dysfunction. These devices decrease left atrial pressure by shunting blood into the right atrium. Normal right heart flow is characterized by a predominant vortex formation in the right atrium, which then enters the right ventricle as a direct flow that preserves kinetic energy and right ventricular work efficiency. Examining the abnormal right heart blood flow patterns in naturally occurring interatrial shunts using 4-dimensional flow magnetic resonance imaging can improve our understanding of the effects of various interatrial shunt devices currently being investigated for heart failure management.

## Introduction

Heart failure (HF) with preserved ejection fraction (HFpEF) is a clinical syndrome associated with poor quality of life, substantial health care resource utilization, and premature mortality.^1^ Elevations in left atrial (LA) pressure at baseline and/or with exercise are omnipresent in patients with HF, leading to pulmonary congestion, lower functional capacity, reduced quality of life, and worse prognosis. The creation of intracardiac shunts to decompress elevations in LA pressure has emerged as a therapeutic strategy in patients with HFpEF, with many devices currently under investigation.^2^ These devices decrease LA pressure by shunting blood to the right atrium (RA) in response to elevations in LA pressure, which might have implications for the flow pattern seen at the right heart. Therefore, understanding normal and abnormal right heart flow patterns is important, particularly given the potential effects of different shunt technologies. Emerging imaging technologies, such as 4-dimensional flow magnetic resonance imaging (4D flow MRI), enable complex right heart flow assessment in all 3 flow directions simultaneously. In this review, we discuss the various aspects of right heart blood flow patterns, as assessed by 4D flow MRI, in the context of shunt devices under clinical evaluation for patients with symptomatic HFpEF.

## Normal right heart flow patterns

From a mechanical standpoint, the right ventricle (RV) is designed differently than the left ventricle (LV). The continuity between the muscle fibers of the RV and LV functionally binds the ventricles together and represents the anatomic basis for RV free ventricular wall traction caused by LV contraction. This continuity also contributes, along with the interventricular septum and pericardium, to ventricular interdependence or coupling.^3^^,^^4^ In addition, LV contraction plays a significant role because interventricular septal shortening generates 20% to 40% of RV stroke volume.^5^

Blood flow from the caval veins enters the RA in a clockwise, rotational, and organized vortex, with the posterior wall of the RA and predominantly inferior vena cava (IVC) flow playing a role in vortex formation.^6^ This rotational nature of the blood flow during filling and emptying maintains kinetic energy (KE), preserves fluid momentum, and minimizes turbulence, which in turn leads to efficient transfer of flow and energy to the RV (Supplemental Videos 1A and B). Using 4D flow MRI, Parikh et al^7^ previously reported a spectrum of primary flow patterns in the RA of normal healthy individuals. These patterns include vortical, helico-vortical, and helical. Vortical flow is defined as a clockwise forward-turning vortex with the central core composed of IVC flow and the superior vena cava (SVC) flow entrained on the outside. Helico-vortical flow refers to a vortex composed solely of IVC flow with SVC flow passing laterally and twisting around it in a helical fashion. Helical flow occurs when IVC flow passes medially and SVC flow laterally, with no primary vortex, and the two are curled together. Multiple vortices are not commonly seen in healthy subjects.^7^

**Figure 1:**
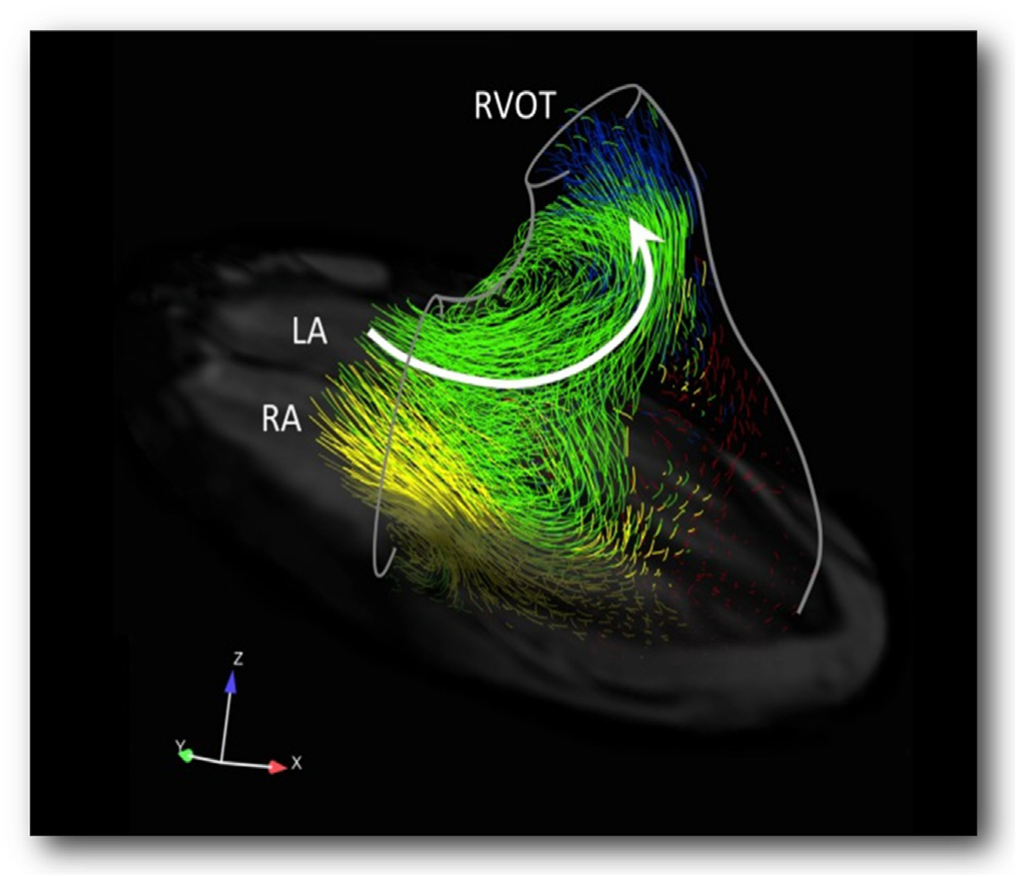
**Normal RV flow patterns as visualized by 4D flow magnetic resonance imaging.** Pathline visualization of RV blood flow during late diastolic filling in a 70-year-old healthy individual with a left ventricular end-diastolic volume index of 61 mL/m^2^. The inflowing blood is channeled toward the outflow tract through the ring vortex (indicated by the white arrow). Different flow components are represented as follows: direct flow (green), retained inflow (yellow), delayed ejection flow (blue), and residual volume (red). Some pathlines in the lateral region of the RV have been omitted to reveal those near the interventricular septum. A semitransparent 4-chamber image provides anatomical orientation. LA, left atrium; RA, right atrium; RV, right ventricle; RVOT, right ventricular outflow tract. Reproduced with permission from Fredriksson et al.^9^

Based on visualized particle tracing through 4D flow MRI, RV blood flow in a single cardiac cycle is divided into 4 different flow components: (1) *direct flow*, blood that enters the RV in diastole and leaves in systole; (2) *retained flow*, blood that enters the RV in diastole but remains in the ventricle during systole; (3) *delayed ejection flow*, blood located at the superior aspect of the RV at the onset of diastole leaving the ventricle in systole; and (4) *residual volume*, blood that remains in the RV for ≥2 cardiac cycles, primarily in the apical and peripheral regions of the RV (Figure 1).^4^^,^^8^^,^^9^

Normal diastolic RV blood flow is dominated by a gradual transition from RV inflow to outflow along the septum through the basal and midventricular segments largely circumventing the apex and is characterized by an asymmetric ring vortex that surrounds the tricuspid inflows. The superior portion of this ring vortex extends into the RV outflow tract near the infundibular septum and contributes to the *direct flow* component of the blood flow, which preserves KE and optimizes RV work efficiency.^10^ The *retained flow* follows an inferior and lateral path contributing to the inferior aspect of the ring vortex. During RV diastasis, flow velocities decrease and the rotation speed of the ring vortex slows followed by acceleration in late diastole. Using 4D intraventricular flow analysis, Fredriksson et al^10^ demonstrated that the *direct flow* component of RV blood flow comprises a substantial portion of total RV volume (∼40%) and possesses significantly larger KE than the other flow components (Supplemental Video 2A). The *direct flow* component favors effective systolic ejection of blood through the RV while maintaining work efficiency. Of note, the RV flow components and KE parameters change significantly with age and sex.^11^

## Abnormal right heart flow patterns

### RV flow pattern in LV disease

The LV plays a significant role in RV systolic flow and pressure generation (via the subepicardial oblique muscle fibers in the LV mediated through the interventricular septum). LV dysfunction, both systolic and diastolic, is a common cause of RV failure.^12^ Using 4D flow MRI assessment, it is possible to determine subtle RV impairment in patients with primary LV disease.^9^^,^^13^ Fredriksson et al^9^ compared healthy controls to patients with diagnosed or suspected chronic ischemic heart disease and assessed changes in RV *direct flow* to study subclinical RV impairment. The study showed that, compared with healthy controls and patients with a lower LV end-diastolic volume index (≤74 mL/m^2^), patients with a higher LV end-diastolic volume index (>74 mL/m^2^) had significantly lower RV *direct flow*, higher *retained inflow*, and higher *delayed ejection flow*, all of which were measured as a percentage of total RV end-diastolic volume. In addition, the KE possessed by the *direct flow* component, measured as a percentage of KE possessed by total RV end-diastolic volume, was also significantly lower in those patients with a higher LV end-diastolic volume index. While the RV diastolic blood flow in healthy controls and patients with a smaller LV end-diastolic volume index appeared to be more organized, patients in the higher end-diastolic volume index group had a less organized flow distribution with higher degrees of mixing between the components. One possible explanation for the altered RV flow dynamics in patients with primary LV disease is the reduced contribution of the interventricular septum (Supplemental Videos 2A and B). The abnormal clockwise flow along the interventricular septum seen in patients with higher LV end-diastolic volume index results in reduced *direct flow* into the RV outflow tract and a lower proportion of RV end-diastolic volume contributing to efficient systolic ejection, which in turn leads to reduced work efficiency of the chamber. In patients with primary LV disease, changes in flow, as assessed by 4D flow MRI, precede any differences seen in conventional MRI and echocardiographic RV indices.

### Other disruptors of normal RV inflow

Other conditions associated with changes in RV inflow patterns include naturally occurring interatrial shunts, pulmonary arterial hypertension, pulmonary regurgitation, and tricuspid regurgitation. In pulmonary arterial hypertension, there is reduced RV *direct flow*, *delayed ejection flow*, reduced peak KE, and increased residual volume.^14^ Similar findings were reported in patients with pulmonary regurgitation and repaired Tetralogy of Fallot.^15^ RV *direct flow* was also shown to be an independent marker of RV function, remodeling, and exercise capacity. In addition, RV work efficiency is a potential new metric to assess RV function. Disruption of normal RV flow patterns correlates with RV remodeling, dysfunction, and exercise intolerance.^15^^,^^16^

Examination of flow patterns in naturally occurring interatrial shunts may provide important insights into different shunt technologies currently under investigation for the management of HF. In the secundum type of atrial septal defects, there is direct left-to-right shunting of blood, which may interfere with the normal vortical blood flow patterns leading to a more turbulent flow of blood in the RA and the RV (Supplemental Video 3). RV chamber work efficiency is dependent on the smooth nonturbulent flow of blood from the RA to RV outflow tract. When the blood flow is more turbulent with increased vorticity, there is a reduction in the *direct flow* momentum component of RV flow, which leads to a possible increase in total KE and decreased RV work efficiency.

**Figure 2:**
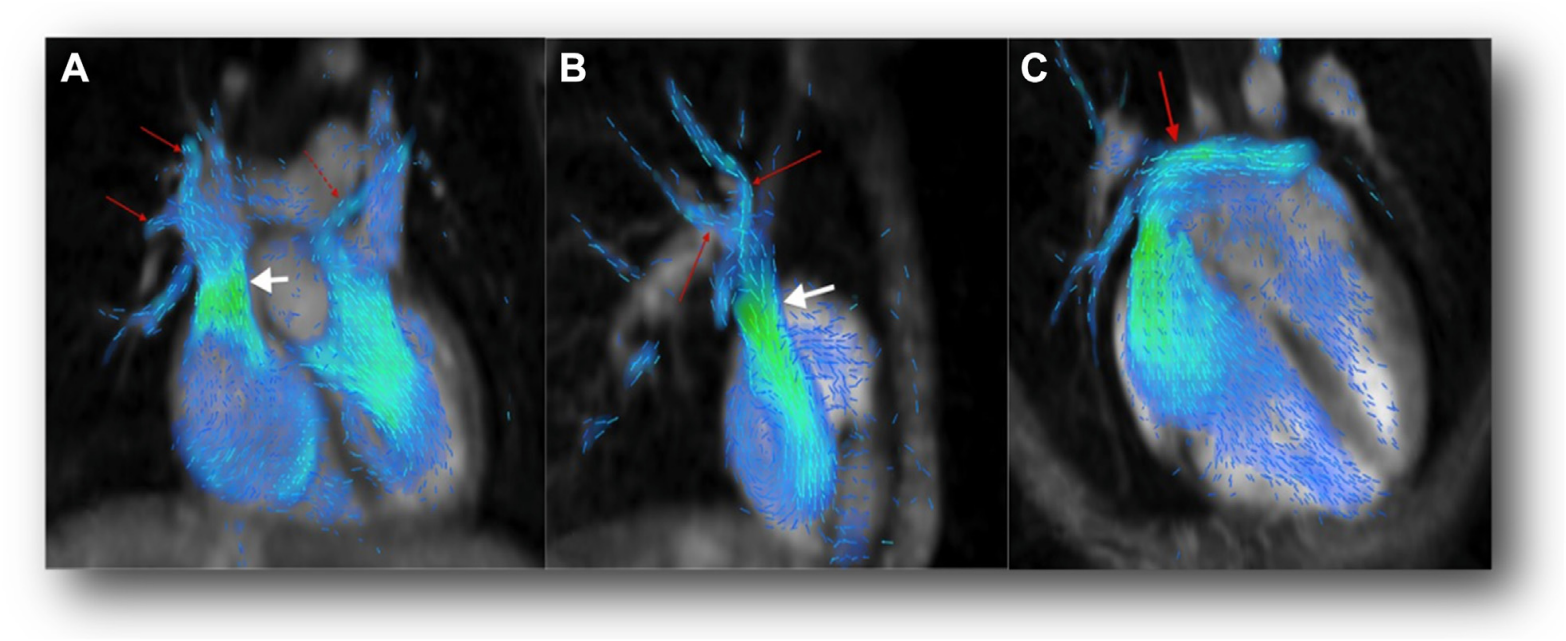
**Partial anomalous pulmonary venous return and a sinus venosus ASD.** Coronal (A) and sagittal (B) views show anomalous right upper pulmonary veins (red arrows) draining to the superior vena cava (white arrow). (C) Oblique four-chamber view shows flow from the left atrium crossing into the right atrium through the atrial septal defect (arrow). Reproduced with permission from Vasanawala et al.^17^

Conversely, nonsecundum atrial septal defects drain primarily into the caval system or coronary sinus and tend to preserve normal RA-RV flow patterns. In Scimitar syndrome, a large subdiaphragmatic pulmonary vein drains blood directly into the IVC. In sinus venosus atrial septal defect, there is an abnormal connection between the atria in the posterior aspect of the interatrial septum close to the junction of the SVC or the IVC and is frequently associated with partial anomalous pulmonary venous return from the right upper lung directly to the SVC. In unroofed coronary sinus, the least common form of interatrial defects, the atrial wall between the coronary sinus and the LA is either partially or completely absent, resulting in left-to-right shunt. In Scimitar syndrome, sinus venosus atrial septal defect, and unroofed coronary sinus (Figure 2, Supplemental Video 4), the abnormal flow reaches the RA via the caval veins or the coronary sinus and may cause less disruption of RA and RV inflows. Nonetheless, regardless of how the LA shunting occurs, the magnitude of the left-to-right shunting can eventually lead to RV volume overload.

## Interatrial shunting in HF

**Figure 3:**
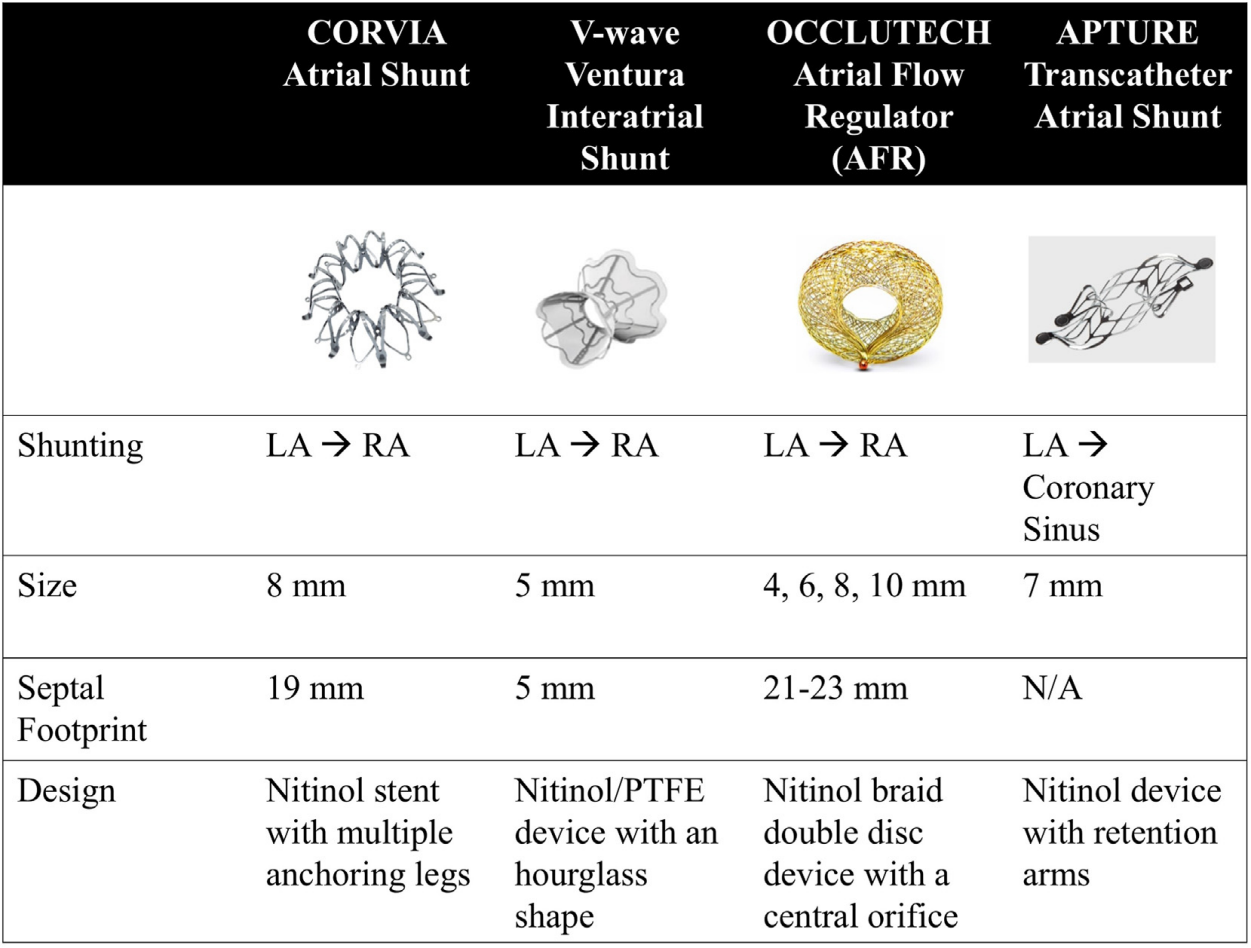
**Interatrial shunt devices in heart failure.** AFR, atrial flow regulator; LA, left atrium; N/A, not applicable; PTFE, polytetrafluoroethylene; RA, right atrium.

Decompressing the LA by controlled shunting of blood to the RA could minimize the rise in LA pressure, which in turn could improve symptoms and clinical outcomes. Several shunting devices are currently under investigation (Figure 3). A review of interatrial shunt therapies is beyond the scope of this review. An excellent review of interatrial shunt therapy for HF can be found in *JSCAI*.^18^ Although preliminary data for interatrial shunt devices in HF showed mixed results, unanswered questions remain. First, it is important to define the HF population more likely to derive benefit and/or harm from LA to RA shunting. Recently, in the RELIEVE-HF trial, the V-Wave device showed significantly lower HF hospitalizations in patients with HF with reduced ejection fraction, but patients with HFpEF had worse overall outcomes compared to a sham procedure, although in the overall HF cohort, there was no difference in the composite of all-cause mortality, left ventricular assist devices/heart transplantation, HF hospitalizations, outpatient worsening HF events, and changes in the Kansas City Cardiomyopathy Questionnaire.^19^ However, in a prespecified analysis stratifying patients based on LV ejection fraction (≤40% and >40%), there were differences in response to treatment, with improved outcomes in patients with HF with reduced ejection fraction (a benefit driven by significant reductions in HF hospitalizations) and worse outcomes in HFpEF patients compared with placebo. The role of cardiac imaging both for patient selection and identification of nonresponders needs further study. Finally, the optimal location, size, and design of the shunt device may have implications for right-sided physiology, which could impact both the efficacy and safety of these technologies. Mild RV dilatation without reduced function was seen in follow-up with the Corvia device in the REDUCE LAP-HF I trial.^20^

Appropriate patient selection is key for shunt therapy because the risk-benefit ratio may differ. Most studies excluded patients with severe RV systolic failure, severe pulmonary hypertension, significant valvular heart disease (especially clinically significant tricuspid regurgitation), and elevated pulmonary vascular resistance (PVR). In fact, the REDUCE LAP-HF trials showed worse outcomes in patients with RA volume index ≥29.7 mL/m^2^ and pulmonary artery systolic pressure of >70 mm Hg at 20 W of exercise. On the contrary, the ALT-FLOW trial did include patients with PVR >3 Wood units (although in small numbers) and did not find any difference in outcomes compared with patients with PVR ≤3 Wood units. Real-world long-term studies in humans are needed to further assess the effect of interatrial shunting on RV size, function, tricuspid regurgitation, and pulmonary artery size.

## Does the location of the interatrial shunt matter for optimal outcomes in HF patients?

**Figure 4:**
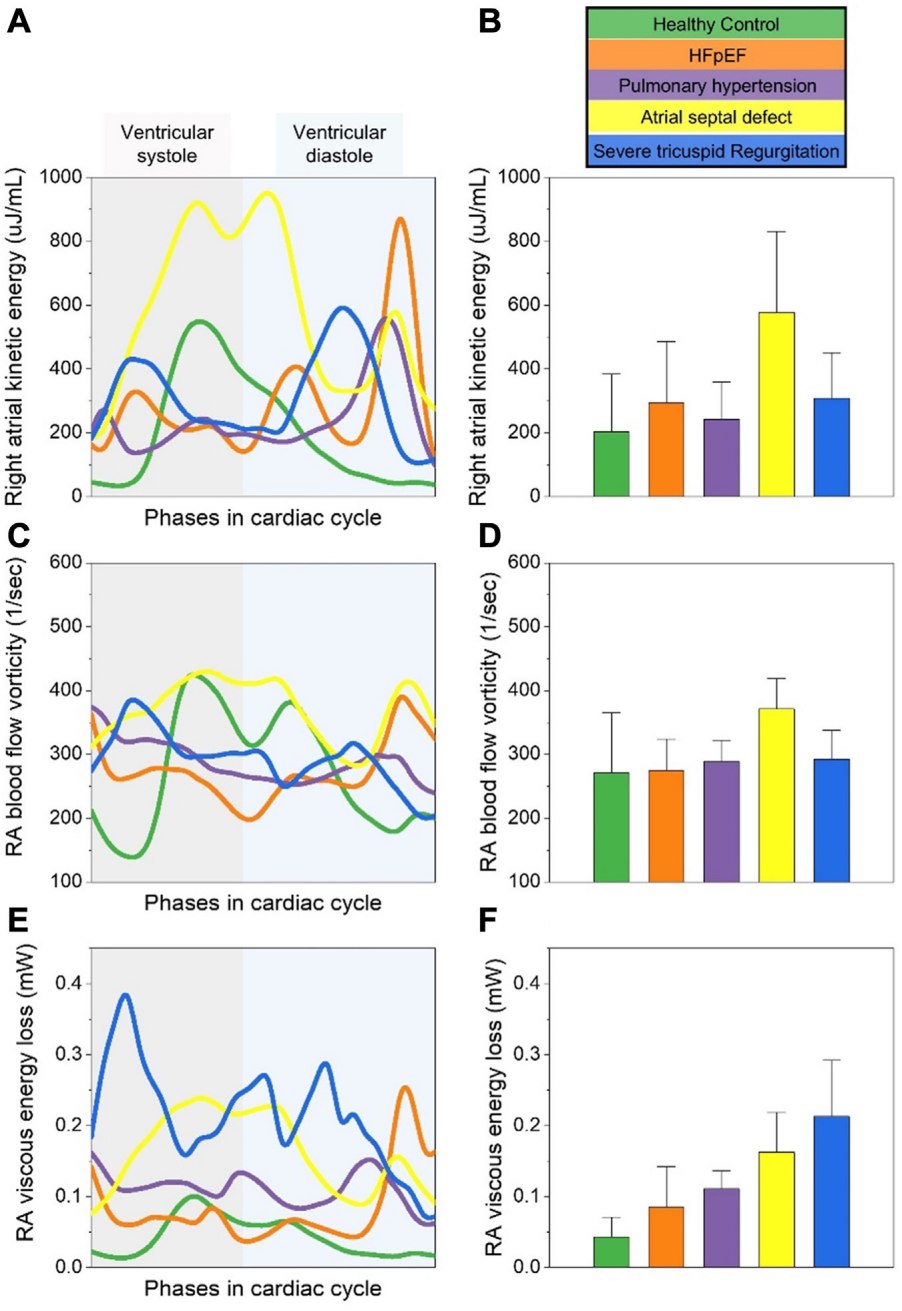
**RA blood flow kinetic energy, vorticity, and viscous energy loss in various cardiac conditions.** (A) RA kinetic energy (μJ/mL) during cardiac cycle phases. (B) Mean RA kinetic energy with SD. (C) RA blood flow vorticity (1/s) over the cardiac cycle phases. (D) Mean RA blood flow vorticity with SD. (E) RA viscous energy loss (mW) during cardiac cycle phases. (F) Mean RA viscous energy loss with SD. Each condition is color-coded: healthy control (green), severe tricuspid regurgitation (orange), HFpEF with AF (purple), pulmonary hypertension (yellow), and atrial septal defect (blue). AF, atrial fibrillation; HFpEF, heart failure with preserved ejection fraction; RA, right atrial.

While most interatrial shunting devices offload the LA by allowing blood flow to pass to the lower-pressure RA, the APTURE shunt device shunts blood from the LA to the coronary sinus. Theoretically, this could result in less disruption of RA and RV vortices and thereby have less effect on the *direct flow* component of RV blood flow. Disruption of RA and RV vortices in direct interatrial shunts (as in secundum atrial septal defects) may lead to higher energy loss and reduced RV filling efficiency, which in turn causes increases RV workload in systole (Central Illustration and Figure 4). Loke et al^16^ compared vorticity and energy loss in healthy controls and patients with RV dilatation secondary to atrial septal defects with significant left-to-right shunt using 4D flow MRI. The study demonstrated that patients with RV dilatation had higher diastolic vorticity in the RV inflow and outflow tracts. In addition, viscous energy loss across the RV outflow tract was greater in patients with RV dilatation than in healthy controls. Despite these differences, both controls and patients with RV dilatation had a dominant “donut”-shaped ring vortex at the tricuspid inflow. Patients with HF are known to have altered RV flow patterns, and further disruption of inflow could contribute to increased RV demand/workload due to its thin wall and reduced contractile reserve, which could potentially translate into less improvement in functional status and exercise capacity. The ongoing ALT-FLOW II trial (NCT05686317) has a prespecified 4D flow MRI substudy protocol with the goals to evaluate changes in right heart flow, quantification of RV size/function, and quantification of shunt magnitude.

**Central Illustration:**
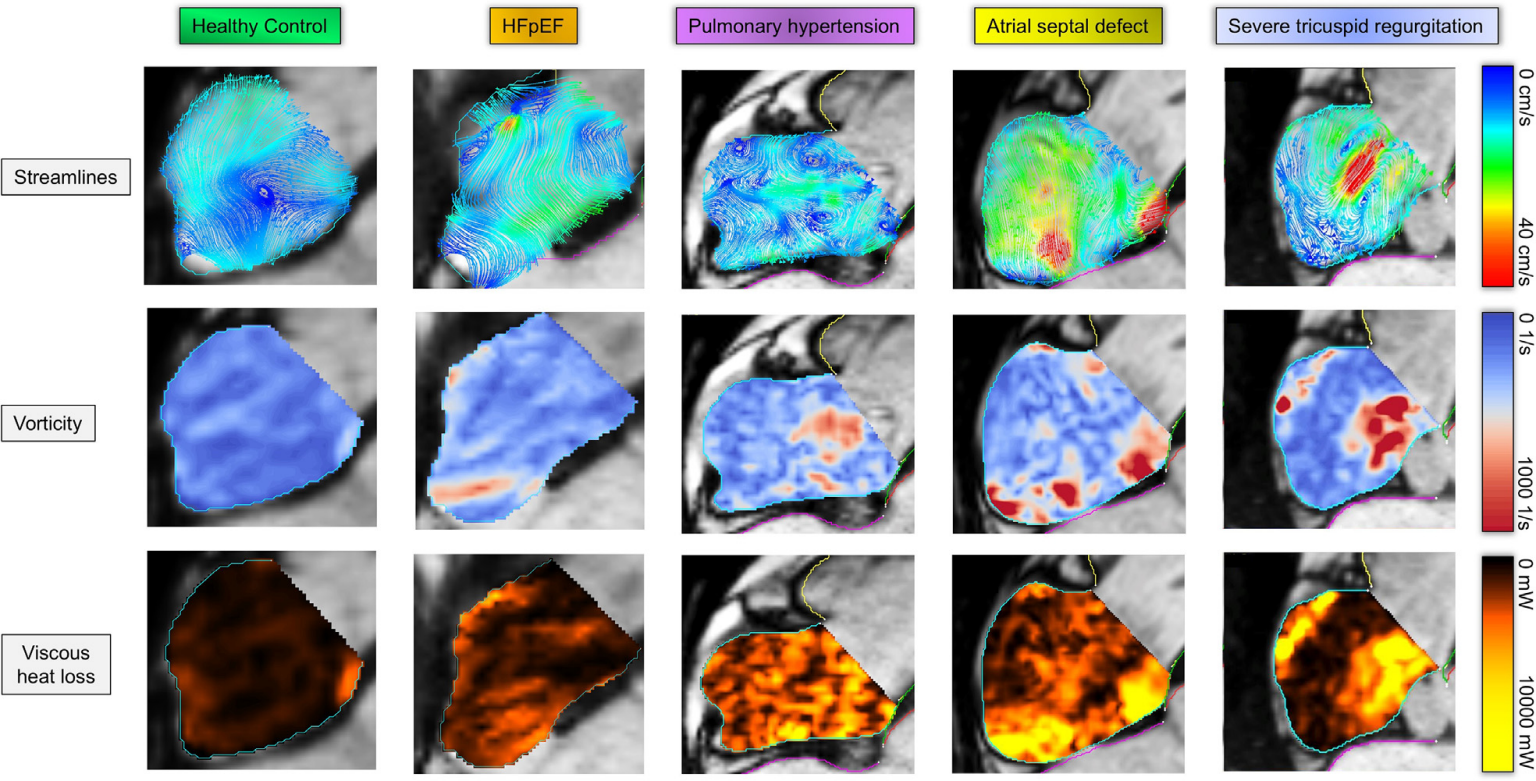
**Visualization of intra-atrial flow patterns, vorticity, and viscous heat loss in various cardiac conditions.** Intra-atrial hemodynamics for a healthy individual (control) and cases of HFpEF, pulmonary hypertension, atrial septal defect, and severe tricuspid regurgitation are shown during atrial filling coinciding with ventricular systole. Rows depict streamlines (top, 0-40 cm/s), vorticity (middle, 0-1000 1/s), and viscous heat loss (bottom, 0-10,000 mW). Healthy individual shows orderly flow, minimal vorticity, and low heat loss. The organized vortical flow appears to reduce heat loss and effectively reduce the overall vorticity. In the HFpEF case, the central organized vortical flow seen in the control is missing, and there is a bit of disorganized flow in streamline visualization, which possibly results in a minor increase in vorticity and heat loss. In a patient with pulmonary hypertension, there is a significant increase in disorganized streamlines in the right atrium. This complements the associated increase in vorticity and heat loss observed. In a case of atrial septal defect, there is a very high velocity signal across the septum in streamline visualization, which appears to result in high vorticity and heat loss. Similar observations are seen in a patient with severe tricuspid regurgitation.

## Conclusion

Normal right heart flow, as assessed by 4D flow MRI, consists of a dominant clockwise ring vortex, and disruption of this vortical flow can result in reduced chamber work efficiency, adverse remodeling and eventually, reduced exercise tolerance. Design differences in shunt devices, including location and size of interatrial shunts and differential shunting to the coronary sinus, may affect the safety and effectiveness of these therapies. Ongoing clinical trials should shed light on the role of shunt devices in the contemporary management of HF, including patient selection.

## Supplementary material

Supplementary Video 1 Normal 4D intracardiac flow (A), right sagittal projection – RA (B). Blood flow from the caval veins into the RA during right ventricular systole is rotational and organized into a vortex formation. IVC, inferior vena cava; RA, right atrium; SVC, superior vena cava.

Supplementary Video 2 4D flow MRI right ventricular flow patterns in patients with heart failure with normal (A) and high (B) left ventricular end-diastolic volume index. Reproduced with permission from Fredriksson et al.^9^

Supplementary Video 3 RA flow in healthy individual (top row) and in a patient with ASD (bottom row). Healthy individual showed preservation of vortex (panel 3), main vortex (dominant red mass) entering tricuspid valve (panel 4) and minimal energy loss (panel 5). In contrast, in the patient with ASD, the main RA vortex is disrupted as the shunt flow cuts across the RA flow (panel 3), resulting in numerous chaotic vortices (panel 4) and a larger energy loss (panel 5). ASD, atrial septal defect; LVEPD, left ventricular end-diastolic pressure; RA, right atrium.

Supplementary Video 4 Unroofed coronary sinus (left), and sinus venosus atrial septal defect (right) showing no significant disruption to the dominant central right atrial vortex. ∗Superior vena cava. Ao, aorta; CS, coronary sinus; LA, left atrium; RA, right atrium; PA, pulmonary artery; RPA, right pulmonary artery; RSPV, right superior pulmonary vein; SVC, superior vena cava. (Reproduced with permission from Sugimori et al, Coronary sinus atrial septal defects in adults over the past 20 years at new Tokyo hospital: case series, Journal of Cardiothoracic Surgery; Mandell et al Diagnosis of a coronary sinus atrial septal defect by 4D flow MRI, Journal of Cardiovascular Magnetic Resonance; Kafka et al, Cardiac MRI and Pulmonary MR Angiography of Sinus Venosus Defect and Partial Anomalous Pulmonary Venous Connection in Cause of Right Undiagnosed Ventricular Enlargement, American Journal of Roentgenology; Vasanawala et al, Congenital heart disease assessment with 4D flow MRI, Journal of Magnetic Resonance Imaging).

## References

[1] Pfeffer M.A., Shah A.M., Borlaug B.A. (2019). Heart failure with preserved ejection fraction in perspective. Circ Res.

[2] Shah S.J., Borlaug B.A., Chung E.S. (2022). Atrial shunt device for heart failure with preserved and mildly reduced ejection fraction (REDUCE LAP-HF II): a randomised, multicentre, blinded, sham-controlled trial. Lancet.

[3] Haddad F., Hunt S.A., Rosenthal D.N., Murphy D.J. (2008). Right ventricular function in cardiovascular disease, part I: anatomy, physiology, aging, and functional assessment of the right ventricle. Circulation.

[4] Bolger A.F., Heiberg E., Karlsson M. (2007). Transit of blood flow through the human left ventricle mapped by cardiovascular magnetic resonance. J Cardiovasc Magn Reson.

[5] Lahm T., Douglas I.S., Archer S.L. (2018). Assessment of right ventricular function in the research setting: knowledge gaps and pathways forward. An official American Thoracic Society research statement. Am J Respir Crit Care Med.

[6] Dewhurst P., Coats L., Parikh J.D., Hollingsworth K.G., Gan L. (2020). The role of flow rotation in the adult right atrium: a 4D flow cardiovascular magnetic resonance study. Physiol Meas.

[7] Parikh J.D., Kakarla J., Keavney B. (2017). 4D flow MRI assessment of right atrial flow patterns in the normal heart - influence of caval vein arrangement and implications for the patent foramen ovale. PLOS ONE.

[8] Eriksson J., Carlhäll C.J., Dyverfeldt P., Engvall J., Bolger A.F., Ebbers T. (2010). Semi-automatic quantification of 4D left ventricular blood flow. J Cardiovasc Magn Reson.

[9] Fredriksson A.G., Svalbring E., Eriksson J. (2016). 4D flow MRI can detect subtle right ventricular dysfunction in primary left ventricular disease. J Magn Reson Imaging.

[10] Fredriksson A.G., Zajac J., Eriksson J. (2011). 4-D blood flow in the human right ventricle. Am J Physiol Heart Circ Physiol.

[11] Zhao X., Tan R.S., Garg P. (2023). Age- and sex-specific reference values of biventricular flow components and kinetic energy by 4D flow cardiovascular magnetic resonance in healthy subjects. J Cardiovasc Magn Reson.

[12] Haddad F., Doyle R., Murphy D.J., Hunt S.A. (2008). Right ventricular function in cardiovascular disease, part II: pathophysiology, clinical importance, and management of right ventricular failure. Circulation.

[13] Barker N., Fidock B., Johns C.S. (2019). A systematic review of right ventricular diastolic assessment by 4D flow CMR. Biomed Res Int.

[14] Zhao X., Leng S., Tan R.S. (2022). Right ventricular energetic biomarkers from 4D flow CMR are associated with exertional capacity in pulmonary arterial hypertension. J Cardiovasc Magn Reson.

[15] Zhao X., Hu L., Leng S. (2022). Ventricular flow analysis and its association with exertional capacity in repaired tetralogy of Fallot: 4D flow cardiovascular magnetic resonance study. J Cardiovasc Magn Reson.

[16] Loke Y.H., Capuano F., Cleveland V., Mandell J.G., Balaras E., Olivieri L.J. (2021). Moving beyond size: vorticity and energy loss are correlated with right ventricular dysfunction and exercise intolerance in repaired Tetralogy of Fallot. J Cardiovasc Magn Reson.

[17] Vasanawala S.S., Hanneman K., Alley M.T., Hsiao A. (2015). Congenital heart disease assessment with 4D flow MRI. J Magn Reson Imaging.

[18] Jagadeesan V., Gray W.A., Shah S.J. (2023). Atrial shunt therapy for heart failure: an update. J Soc Cardiovasc Angiogr Interv.

[19] Stone G.W., Lindenfeld J., Rodés-Cabau J. (Published online September 23, 2024). Interatrial shunt treatment for heart failure: the randomized RELIEVE-HF trial. Circulation.

[20] Feldman T., Mauri L., Kahwash R. (2018). Transcatheter interatrial shunt device for the treatment of heart failure with preserved ejection fraction (REDUCE LAP-HF I [Reduce Elevated Left Atrial Pressure in Patients With Heart Failure]): a phase 2, randomized, sham-controlled trial. Circulation.

